# Kidney interstitial edema as the central pathophysiological mechanism of NSAKI: a call for reappraisal

**DOI:** 10.1093/ckj/sfaf271

**Published:** 2025-08-30

**Authors:** Osamu Uemura

**Affiliations:** Ichinomiya Medical Treatment & Habilitation Center, Ichinomiya, Aichi, Japan

To the Editor,

Nephrotic syndrome-induced acute kidney injury (NSAKI) is a distinct but often underrecognized clinical entity, especially in children with MCD or FSGS. Despite research on nephrotic syndrome and AKI, the pathophysiological link remains unclear. We propose that interstitial edema-driven kidney ischemia is the central mechanism of NSAKI.

The hallmark of nephrotic syndrome—hypoalbuminemia—leads to a decrease in plasma oncotic pressure. This shift promotes transcapillary fluid movement into the interstitium, causing generalized edema, including in the kidney interstitium. As kidney tissues become edematous, intrarenal pressure rises, compressing both glomerular and peritubular capillaries, leading to ischemia. Systemic hemodynamics may seem normal or overloaded, yet kidney perfusion remains low.

A landmark study by Vande Walle *et al.* observed preserved renal plasma flow with decreased glomerular filtration rate and filtration fraction (FF) in children with MCD and oliguric AKI, suggesting elevated Bowman's space pressure secondary to interstitial edema [1]. This dissociation highlights a localized rather than systemic process. In addition, Loghman-Adham's review found acute tubular necrosis (ATN) in nearly half of NSAKI cases, often with protracted recovery over months [2]. These findings support persistent, low-grade, and regionally restricted ischemia rather than generalized vascular collapse.

Furthermore, low fractional excretion of sodium (fractional excretion of sodium; FENa <1%) is paradoxically observed in NSAKI patients, even with apparent fluid overload. Traditional interpretations attribute this to enhanced sodium reabsorption at the distal nephron or blunted natriuretic peptide responses [3, 4]. However, these theories describe secondary adaptations and fail to explain why sodium reabsorption increases or natriuretic peptide responses diminish as primary processes, nor do they account for reduced FF or histologic ATN. We argue that physical compression from interstitial edema provides a more comprehensive mechanistic explanation.

It is critical to recognize the dynamic and evolving fluid compartments in nephrotic syndrome. Initially, most pediatric cases reflect an “underfilling” state, where hypoalbuminemia causes plasma volume depletion. Yet, as interstitial edema progresses, compensatory mechanisms such as RAS activation restore intravascular volume, even leading to overfilling. At this stage, kidney congestion and increased interstitial pressure may reduce capillary flow and oxygen delivery, despite preserved or elevated systemic pressures.

Histologically, interstitial edema, often with ATN, is confirmed in NSAKI biopsies, and massive proteinuria itself can injure proximal tubules, predisposing to severe AKI. These may reflect ischemia worsened by intrarenal hypertension. Indeed, ischemia-reperfusion injury is known to induce inflammatory cascades and epithelial cell apoptosis, mechanisms also implicated in prolonged kidney dysfunction observed in NSAKI.

Therapeutically, this pathophysiological understanding demands a paradigm shift. Relieving intrakidney congestion is essential. Albumin-furosemide combination therapy has shown superior efficacy in increasing diuresis and sodium excretion compared to diuretics alone [5]. However, both agents pose nephrotoxic risks and must be used judiciously. In resistant cases, dialysis or ultrafiltration may be needed to remove fluid while preserving perfusion.

Recovery depends on achieving remission. In adults, NSAKI occurs in ∼40%, often in steroid-resistant or frequently relapsing disease. Targeted therapy and early biopsy may help. Clinicians should monitor for complications like PRES, often triggered by rapid volume shifts.

While alternative hypotheses such as primary sodium retention or hormonal dysregulation exist, they do not fully align with the pathologic and hemodynamic observations in NSAKI. Edema-induced ischemia offers a unifying framework that explains reduced FF, histologic ATN, paradoxical sodium retention, and delayed recovery.

We urge the nephrology community to reconsider NSAKI pathophysiology. Imaging is informative: kidney enlargement appears in about half of both adult and pediatric cases reported in case series. Studies using kidney imaging, biomarkers, and pathology are needed. Recognizing interstitial edema as the core insult may lead to earlier identification and more effective treatment strategies.

## Data Availability

No new data were generated or analyzed in support of this research.
